# Loganin Exerts Sedative and Hypnotic Effects via Modulation of the Serotonergic System and GABAergic Neurons

**DOI:** 10.3389/fphar.2019.00409

**Published:** 2019-04-24

**Authors:** Rui Shi, Yan Han, Yu Yan, Hao-Yi Qiao, Jun He, Wen-Wen Lian, Cong-Yuan Xia, Ting-Li Li, Wei-Ku Zhang, Jie-Kun Xu

**Affiliations:** ^1^School of Life Sciences & School of Chinese Medicine Sciences, Beijing University of Chinese Medicine, Beijing, China; ^2^Institute of Clinical Medical Sciences & Department of Pharmacy, China-Japan Friendship Hospital, Beijing, China; ^3^School of Chinese Medicine Sciences, Heilongjiang University of Chinese Medicine, Harbin, China

**Keywords:** loganin, sedative, hypnotic, serotonergic system, GABAergic neurons

## Abstract

Corni fructus, the fruit of *Cornus officinalis* Sieb. et Zucc., has been used as a tonic for the kidney in China for thousands of years. Loganin is one of the major constituents derived from Corni fructus. In this study, we revealed the sedative and hypnotic activity of loganin and investigated its mechanisms for the first time. Pentobarbital-induced sleep test and insomnia mice models [induced by caffeine and p-chlorophenylalanine (PCPA)] were used for the assessment of sedative and hypnotic effects of loganin. It was found that loganin (20–50 mg/kg) exerted sedative effect in normal mice. Loganin exhibited hypnotic effect by increasing sleep onset and sleep duration in pentobarbital-treated mice, recovering PCPA-induced insomnia and exerting synergistic hypnosis effect with 5-HTP. In addition, electroencephalograph (EEG) and electromyography (EMG) recordings of rats showed that loganin (35 mg/kg) prolonged the ratio of non-rapid eye movement (NREM) sleep and shortened wakefulness significantly, further immunohistochemistry showed that loganin (35 mg/kg) increased c-Fos expression in GABAergic neurons of rats in the ventrolateral preoptic nucleus (VLPO). The levels of norepinephrine (NE), dopamine (DA), serotonin (5-HT) and its metabolite were measured in the hippocampus, prefrontal cortex and striatum of mice, 1 h after loganin (35 mg/kg) treatment. 5-HT, 5-HIAA/5-HT, DA, and DOPAC were decreased significantly in the prefrontal cortex. In conclusion, these results indicated that loganin produced beneficial sedative and hypnotic activity, which might be mainly mediated by modification of the serotonergic system and GABAergic neurons.

## Introduction

Insomnia is referred to as continuous difficulty in initiating or maintaining sleep, which can induce extreme medical and psychiatric disorders (Cao et al., 2016). It was reported that 27 percent of people in the world suffering from insomnia (Doghramji, 2006), and approximately 3–10 percent of people would frequently depend on hypnotics to overcome insomnia by 2050 (Chu et al., 2007). Clinically, benzodiazepines are the most extensively used drug for insomnia. Nevertheless, serious side effects are always accompanied with their treatment, including drug dependence, drug tolerance, rebound insomnia, and amnesia (Griffiths et al., 1986; Bocca et al., 1999). Zolpidem and zopiclone, a new type of hypnotic, also show some undesirable side effects. Therefore, novel drug for insomnia with better effect and less side effect deserves more effort, and novel herbal ingredients in traditional Chinese medicine might be a better choice for drug discovery (Griffiths et al., 1986).

**FIGURE 1:**
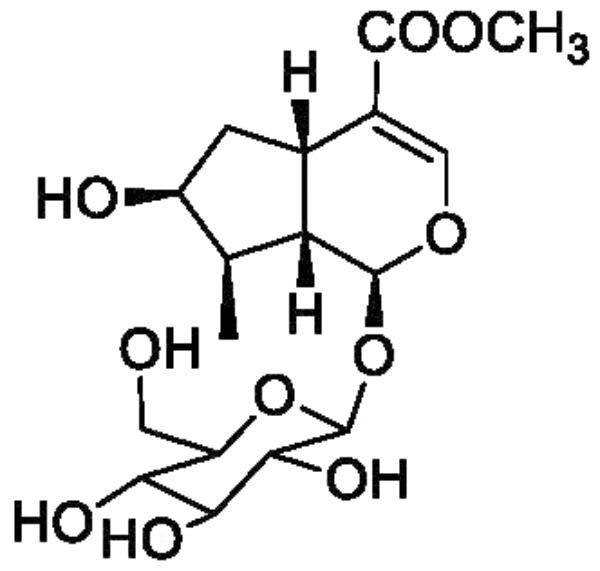
Chemical structure of loganin.

Corni fructus, the fruit of *Cornus officinalis* Sieb. et Zucc. (Cornaceae), has long been used to nourish the liver and kidney and treat diabetes and other diseases in China, Korea, and Japan (Lee et al., 2009). Loganin (Figure 1), a major iridoid glycoside isolated from Corni fructus, has been reported to show neuroprotective activity, immunity regulatory activity, and lipid metabolism regulatory activity, and further exhibit preventative and treatment effects in diabetes, tumors, inflammation, and Alzheimer’s disease (Hui-Jun et al., 2003; Frédérich et al., 2004; Lee et al., 2009). In addition, loganin has been proved to pass through the blood-brain barrier and reaches the brain (Li et al., 2010), which might be the basis of its neuroprotective activity. However, the sedative and hypnotic effects of loganin have not yet been reported.

Monoamine neurotransmitters and their metabolites play a significant role in the nervous system and have also been associated with insomnia. Mammalian sleep is composed of non-rapid eye movement (NREM) and rapid eye movement (REM) sleep alternation (Weber, 2017). The ventrolateral preoptic nucleus (VLPO) cluster is a necessary component of sleep circuitry, the discharge rate of which increases just before or at the transition from wakefulness to NREM sleep and decreases prior to the transition from NREM or REM sleep to wakefulness (Szymusiak et al., 1998). To the best of our knowledge there is no reported mechanism of sedative and hypnotic effects of loganin is related to monoamine neurotransmitters and VLPO neurons.

In this paper, we investigated the sedative activity of loganin using locomotor activity test and then detected the hypnotic effect of loganin in pentobarbital-induced sleep test and insomnia mice models [induced by caffeine and p-chlorophenylalanine (PCPA)]. In addition, we detected the effects of loganin on sleep architecture in free-moving rats through electroencephalograph (EEG) and electromyography (EMG) recording. Furthermore, we evaluated the concentration of monoamine neurotransmitters and their metabolites using high-performance liquid chromatography with electrochemical detection (HPLC-ECD) and assessed the ratio of c-Fos-positive glutamic acid decarboxylase (GAD) neurons in VLPO, the main brain region triggers NREM sleep (Cao et al., 2016), trying to explain the mechanism of loganin.

## Materials and Methods

### Animals

Adult male ICR mice (20 ± 2 g) and adult male Sprague-Dawley rats (260–280 g) were purchased from Vital River Laboratories (Beijing, China). The animals were housed in acrylic cages (45 cm × 60 cm × 25 cm) with water and food available *ad libitum* under an artificial 12 h light/dark cycle (light from 8:00 am to 8:00 pm) in a sound-proof room, and the temperature (24 ± 2°C) the humidity (55 ± 15%) were controlled.

Experiments were conducted in compliance with the National Institutes of Health Guide for the Care and Use of Laboratory Animals of China and were approved by the Animal Care Committee of China-Japan Friendship Hospital. Every effort was made to minimize the use of animals and any pain or discomfort.

### Drugs

Commercially available drugs used in this study were purchased: pentobarbital (Sigma-Aldrich), Estazolam tablet (1.0 mg/60 mg; Shandong Xinyi Pharmaceutical Co., Ltd., Shandong, China), anhydrous sodium acetate (Thermo Fisher Scientific), citric acid (Coolaber), sodium octane sulfonate (Adamas-beta), EDTA-2Na (Beijing Biodee Biotechnology Co., Ltd., Beijing, China), KCl (Thermo Fisher Scientific), perchlorate (Tianjin Damao Chemical Reagent Factory, Tianjin, China). HPLC-grade water and methanol were obtained from Fisher Scientific (Thermo Fisher Scientific). 3,4-Dihydroxybenzylamine hydrobromide (DHBA), norepinephrine (NE), 3,4-dihydroxy-phenyl acetic acid (DOPAC), dopamine (DA), 5-hydroxyindoleacetic acid (5-HIAA), homovanillic acid (HVA), and serotonin (5-HT) were purchased from Sigma-Aldrich. PCPA, caffeine, and 5-HTP were obtained from Melonepharma Co., Ltd. (Dalian, China).

### Extraction, Isolation, and Structural Elucidation of Loganin

Corni fructus (63.0 kg) collected in Foping, Shaanxi province, China in November 2013 were refluxed twice with hot water (630 L, 100°C) for 2 h. The solvent was concentrated to afford 28.9 kg of crude extract, which was separated via a macroporous adsorption resin (D101) column eluting with H_2_O, 30% ethanol, 55% ethanol, and 95% ethanol. The 30% ethanol residue (2.9 kg) was separated on a silica gel column with CHCl_3_–CH_3_OH (100:0 to 1:1) to give five fractions (A–E). Fraction B (1.0 kg) was dissolved in CH_3_OH, and placed at the room temperature for 24 h. The sediment (300 g) was repeatedly dissolved in ethanol and ethyl acetate to recrystallize to obtain loganin (200 g).

The NMR data of loganin are as follows: ^1^H-NMR (CD_3_OD, 400 MHz), δ: 5.28 (1H, d, 4.4 Hz, H-1), 7.40 (1H, s, H-3), 3.37 (1H, m, H-5), 1.63 (1H, m, H-6), 1.88 (1H, m, H-6), 3.67 (1H, m, H-7), 2.05 (1H, m, H-8), 2.25 (1H, m, H-9), 1.11 (3H, d, 7.2 Hz, H-10), 3.70 (3H, s, COOCH_3_), 4.66 (1H, d, 8.0 Hz, H-1′), 3.10∼3.39 (m, H-2′∼H-5′), 3.66 (dd, 1H, 1.2, 11.6 Hz, H-6′), 3.89 (dd, 1H, 1.2, 11.6 Hz, H-6′); ^13^C-NMR (CD_3_OD, 100 MHz), δ: 97.7 (C-1), 152.2 (C-3), 114.1 (C-4), 32.2 (C-5), 42.7 (C-6), 75.1 (C-7), 42.2 (C-8), 46.5 (C-9), 13.5 (C-10), 169.6 (C-11), 51.7 (−OCH_3_), 100.1 (C-1′), 74.8 (C-2′), 78.4 (C-3′), 71.6 (C-4′), 78.1 (C-5′), 62.8 (C-6′). The data are identical to those of the reference (Jeong et al., 2012). The purity of loganin is 99% by HPLC detection.

### Treatments

The animals were adapted to laboratory conditions 7 days before the behavioral study and were maintained in the laboratory until the study was completed.

For oral administration (0.2 ml/10 g for mouse, 1.0 ml/100 g for rat, volume/body weight, i.g.). Animals were orally administered loganin (5, 20, and 50 mg/kg for mouse, 35 mg/kg for rat) and estazolam (0.15 mg/kg for mouse, 0.1 mg/kg for rat), which were suspended in distilled water and administered 25 min before pentobarbital administration (Qiao et al., 2017).

In the pentobarbital-induced sleep test, 45 mg/kg pentobarbital (i.p.) was used as the hypnotic dose (sleep onset = 100%), and 23 mg/kg (i.p.) was used as the subhypnotic dose (sleep onset <10%).

Caffeine (7.5 mg/kg, i.p.), PCPA (300 mg/kg, s.c.) and 5-HTP (2.5 mg/kg, i.p.), were suspended in physiological saline. For the PCPA-induced insomnia, PCPA was injected in mice between 08:00 am and 09:00 am, 24 h prior to the pentobarbital injection. For the caffeine-induced insomnia model, mice received an injection of caffeine between 1:00 pm and 1:30 pm, with the caffeine treatment 30 min prior to the pentobarbital injection. 5-HTP was injected 15 min prior to pentobarbital administration (i.p.).

All drugs were prepared before the experiment each day.

### Behavioral Tests

The behavioral tests were conducted in a soundproof room. The experimenters were blind to the treatment.

### Locomotor Activity Test

Experiments were carried out between 1:00 pm and 5:00 pm. The spontaneous locomotor activity of mice was used to investigate the sedative effects.

A mouse was placed in 1 of 4 boxes in an apparatus (ZIL-2, Institute of Materia Medica, Chinese Academy of Medical Sciences, China). The locomotor activities of the mice were recorded 5, 30, 60, 120, 180, and 240 min after loganin and estazolam injection. Mice were acclimated to the boxes for 2 min, and the locomotor activity of each mouse was monitored for 5 min with activity counts recorded. After each testing session, the enclosures were cleaned with 75% ethanol and water (Xiao-Yu et al., 2014).

### Pentobarbital-Induced Sleep Test

Pentobarbital-induced sleep test in mice were performed, which is a behavioral method to evaluate sedative-hypnotic activity (Masur et al., 1971). Experiments were carried out between 1:00 pm. and 5:00 pm. Mice were placed on their back and observed for sleep onset following pentobarbital injection. The criterion for a mouse to fall asleep was that the mouse lost the righting reflex and remained on their back for more than 1 min. The loss of righting reflex was defined as a failure of the mouse to right itself within 10 s after being placed on its back. The time between the administration of pentobarbital and the loss of righting reflex was recorded as the time to sleep onset, while the time from the loss of righting reflex to recovery was recorded as sleep duration. In the subhypnotic pentobarbital test, the percentage of mice that fell asleep was calculated as follows: Sleep onset (%) = No. falling asleep/Total No. × 100%.

### EEG and EMG Recordings and Analysis

Under deep anesthesia with pentobarbital sodium (45 mg/kg, i.p.), rats were placed in a stereotaxic apparatus to position their bodies. Briefly, two stainless steel screws attached to an insulated wire were implanted in the skull over the frontal parietal cortex for EEG recordings. One was placed approximately 2 mm anterior and 2 mm to the right of bregma; another was placed approximately 3 mm posterior and 2 mm to the left of bregma. A pair of wire electrodes was inserted into the neck muscle to record the EMG. The connecting wires attached to the electrodes were welded to a six-pin connector and attached to the skull, secured with dental cement. After surgery, the rats were given antibiotics for 3 days, placed in separate cages and allowed to recover for a week. The recordings were carried out immediately after intragastric administration of control drug or loganin (35 mg/kg). The signals were amplified and sampled at 200 Hz by a polygraph (Model MP 150; BIOPAC Systems, Goleta, CA, United States). The signals were filtered (EEG, 0.5–30 Hz; EMG, 16–128 Hz), then digitized at a sampling rate of 128 Hz and recorded using AcqKnowledge software (BIOPAC Systems, Inc., United States). The analysis was based on the standard criteria embedded in SleepSign v.3.0 software (BIOPAC Systems). Each epoch was characterized as follows: wakefulness (low amplitude EEG activity, high-voltage EMG activity), NREMS (high amplitude slow or spindle EEG activity, low-amplitude EMG activities), and REMS (low-voltage EEG featured theta wave and very low EMG activities). Total sleep time was calculated as non-rapid eye movement (NREM) time plus rapid eye movement (REM) time. the details of the data parameters with four frequency ranges as follows: the delta band (0.5–4 Hz), theta band (4–8 Hz), alpha band (8–13 Hz), and beta band (13–30 Hz) (Cui et al., 2012). The data were analyzed by automatic scoring and manually checked for possible errors in the automatic scoring. Recordings were performed during the normal wake period of mice to better observe the hypnotic effect of loganin. The power of each frequency band was averaged and expressed as a percent of the total power within the frequency range of 0.5–30 Hz (Qiao et al., 2017).

### c-Fos Immunohistochemistry Analysis

One hour after loganin (35 mg/kg) administration (12:00 am), the rats were anesthetized with pentobarbital (45 mg/kg, i.p.) and the heart was perfused with saline, followed by ice-cold 4% polyformaldehyde (PFA) in 0.01 M phosphate buffered saline (PBS) (pH 7.4). Then, their brains were removed, post-fixed in 4% PFA for 24 h at 4°C and immersed in 30% sucrose at 4°C until they sank. VLPO was obtained by mapping the brains of mice (Konsman, 2003). After that, the frozen sections were cut into pieces of 30 μm thickness on the coronal plane using a cryotome. Sections were double immunofluorescence labeled using methods previously described with some modification (Sherin et al., 1998). Each section was double-immunostained with nucleus-specific neurotransmitter markers labeled green and c-Fos labeled red. The sections were examined using an Olympus IX71 fluorescence microscope (Olympus Corporation, Tokyo, Japan).

The c-Fos positive ratio was calculated as follows:

$$\begin{array}{c} \mathrm{The c-Fos positive ratio}\\ \;=100\times (\mathrm{No. c-Fos}(+)-\mathrm{neurotransmitter synthesizing}\\ \;\mathrm{enzyme}(+)\mathrm{cells/No. c-Fos}(\pm )-\mathrm{neurotransmitter}\\ \;\mathrm{synthesizing enzyme}(+)\mathrm{cells}). \end{array}$$

The average number of c-Fos (+) cells in the nuclei of both hemispheres was taken for statistical analysis.

### Monoamine Neurotransmitters Content Analysis by HPLC-ECD

The rats were sacrificed 1 h after loganin administration (12:00 am). The process and conditions of tissue preparation and monoamine testing were conducted as described with little modification (Zhang et al., 2014). Hippocampus, frontal cortex, and striatum were obtained according to the mouse brain atlas (Konsman, 2003). Brain neurotransmitters and metabolites were analyzed using HPLC-ECD.

**FIGURE 2:**
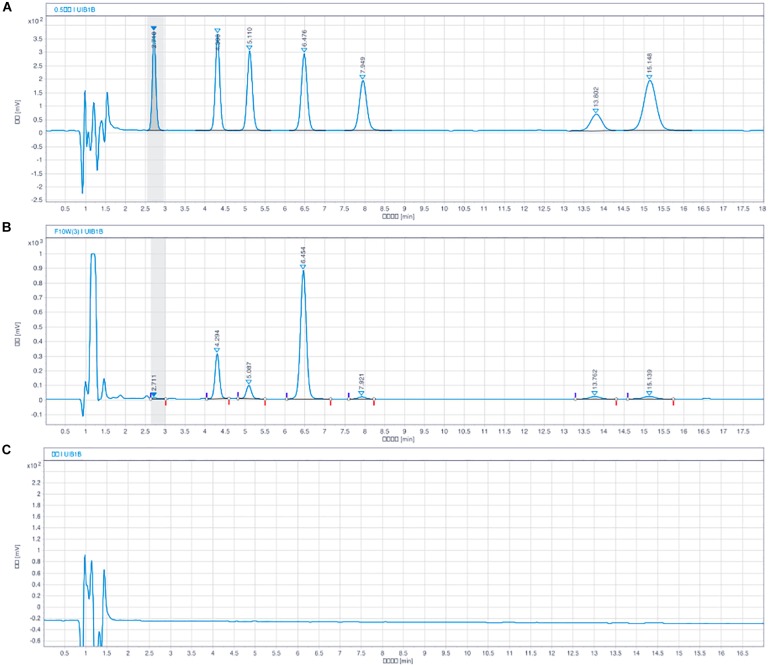
Chromatograms of monoamine neurotransmitter with HPLC-ECD assay. NE (*t*_R_ = 2.7 min), DHBA (*t*_R_ = 4.3 min, internal standard), DOPAC (*t*_R_ = 5.1 min), DA (*t*_R_ = 6.4 min), 5-HIAA (*t*_R_ = 7.9 min), HVA (*t*_R_ = 13.5 min), 5-HT (*t*_R_ = 15.7 min). **(A)** Blank solution, **(B)** Solution of mixed standard substances, and **(C)** Sample solution of rat brain.

As shown in Figure 2, the peak areas in the sample chromatograms and the reference standards were determined by the interior standard method. Reference standards using solutions of NE, 3, 4-dihydroxybenzylamine hydrobromide (DHBA, interior label), DA, DOPAC, 5-HT, HVA, and 5-HIAA. The results are expressed in nanograms per milligram of fresh tissue.

### Statistical Analysis

The results were expressed as the mean ± SEM. For multiple comparisons in the pentobarbital-induced sleep test, data were analyzed using one-way analysis of variance (ANOVA) was used followed by the Students-Newman-Keuls (SNK) test. One-way ANOVA followed by the Least Significance Difference test was applied to the results of EEG and EMG recordings. For comparisons between two-group data, the unpaired Student’s *t*-tests was used. For the subhypnotic dose of pentobarbital test, a χ^2^ test was used to compare the number of mice that fell asleep. Differences were considered statistically significant at ^∗^*P* < 0.05.

## Results

### Sedative Effect of Loganin in Normal Mice

**FIGURE 3:**
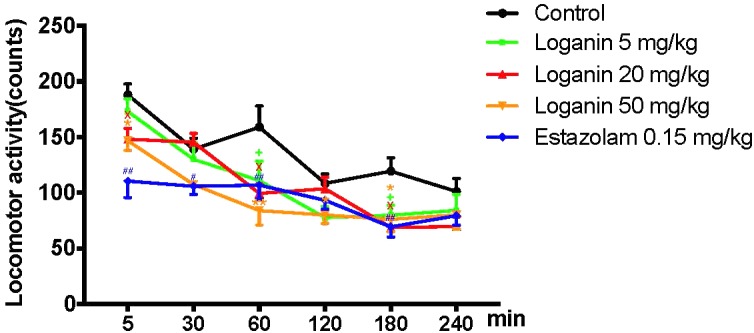
Inhibitory effect of loganin on locomotor activity in normal mice. 5–240 min after administration of loganin (5, 20, 50 mg/kg, i.g.) or estazolam (0.15 mg/kg, i.g.), spontaneous locomotor activity was monitored. Data are presented as means ± SEM (*n* = 12). ^+^*P* < 0.05, loganin 5 mg/kg, vs. the control group; ^X^*P* < 0.05, loganin 20 mg/kg, vs. the control group; ^∗^*P* < 0.05, ^∗∗^*P* < 0.01, loganin 50 mg/kg vs. the control group; ^#^*P* < 0.05, ^##^*P* < 0.01, estazolam 0.15 mg/kg, vs. the control group (one-way ANOVA followed by Student-Newman-Keuls test).

To explore whether the excitability of the central nervous system was affected by loganin, locomotor activity in normal mice was assessed. As shown in Figure 3, locomotor activity was recorded at 5, 30, 60, 120, 180, and 240 min after drug administration. And the locomotor activity of the 5 groups gradually decreased. Compared with the control group, the locomotor activity of loganin (20 and 50 mg/kg) decreased significantly from 5 min (^∗^*P* < 0.05 at 20 and 50 mg/kg) to 180 min (^∗^*P* < 0.05 at 20 and 50 mg/kg), which demonstrated that the sedative effect of loganin appeared as early as 5 min and last for 180 min. Estazolam also showed a similar inhibition on locomotor activity in normal mice (^∗^*P* < 0.01 at 5 and 180 min).

### Onset and Duration of Sleep in Pentobarbital-Induced Sleep Test

To investigate the hypnotic activity of loganin, the effect of loganin on the onset and duration of pentobarbital-induced sleep in mice was evaluated. In mice treated with subhypnotic doses of pentobarbital (23 mg/kg, i.p.), loganin (5, 20, and 50 mg/kg, i.g.) prominently increased sleep onset in mice in a dose-dependent manner (^∗^*P* < 0.05 at 5, 20, and 50 mg/kg, Table 1), compared with the vehicle group. Loganin at 20 and 50 mg/kg increased the sleep onset to 60%, which was slightly lower than the positive control estazolam at 1 mg/kg.

**Table 1:** Effect of loganin on sleep onset of mice induced by subhypnotic dosage of pentobarbital.

Groups	Dosage (mg/kg, i.g.)	No. of falling asleep/total	Sleep onset (%)
Vehicle	–	1/25	4.0
Estazolam	0.15	25/25	100.0^∗^
Loganin	5	9/25	36.0^∗^
	20	15/25	60.0^∗^
	50	15/25	60.0^∗^

*Twenty five minutes after administration of distilled water, estazolam, and loganin; pentobarbital (23 mg/kg, i.p.) was given to mice. The number of mice falling asleep was recorded (n = 12), ^∗^P < 0.05, vs. vehicle (χ^2^ test).*

**FIGURE 4:**
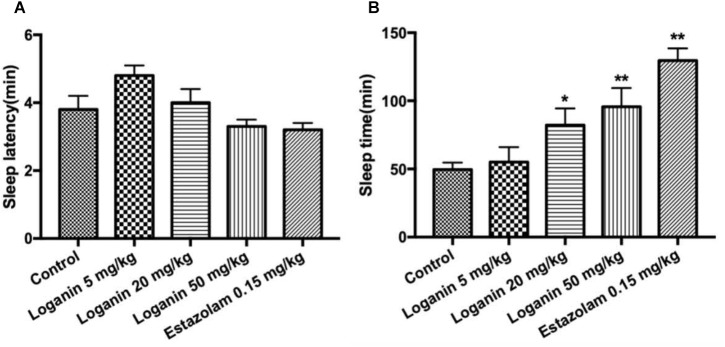
Effect of loganin on the hypnotic response to pentobarbital-induced sleep in mice. The sleep latencyponse to pentobarbital-induced sleep in mice. The sleep latency **(A)** and the sleep time **(B)** were measured. Data are presented as mean ± SEM (*n* = 12). ^∗^*P* < 0.05, ^∗∗^*P* < 0.01, vs. the control group (one-way ANOVA followed by Student-Newman-Keuls test).

In mice with a hypnotic dose of pentobarbital (45 mg/kg, i.p.) treatment, loganin at 50 mg/kg only showed a tendency to reduce sleep latency with no significance (Figure 4A). In terms of sleep duration, loganin at 20 and 50 mg/kg significantly prolonged sleep duration (^∗^*P* < 0.05 at 20 mg/kg; ^∗∗^*P* < 0.01 at 50 mg/kg, Figure 4B) to a slightly lower extent than the positive control estazolam at 0.15 mg/kg.

### Caffeine-Induced Sleep Disturbance in Pentobarbital-Induced Sleep Test

**FIGURE 5:**
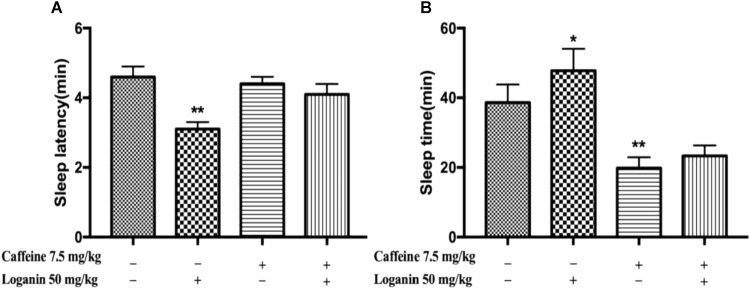
Effects of loganin on caffeine-induced insomnia in pentobarbital-treated mice. The sleep latency **(A)** and sleep time **(B)** were assessed. Data are presented as mean ± SEM (*n* = 10). ^∗^*P* < 0.05, ^∗∗^*P* < 0.01, vs. vehicle (one-way ANOVA followed by Student-Newman-Keuls test).

Mice treated with caffeine, which have been used as an insomnia model, was used to explore the effect of loganin on sleep disturbances in mice. As shown in Figure 5, caffeine did not affect the sleep latency (Figure 5A), but decreased the sleep time significantly (*P* < 0.01, Figure 5B). Loganin at 50 mg/kg attenuated sleep latency (Figure 5A). However, it only showed a tendency to decrease sleep latency when combined with caffeine. Loganin at 50 mg/kg increased sleep time (Figure 5B) compared with the vehicle group, but it had no effect on the decreased sleep time induced by caffeine in pentobarbital-treated mice.

### PCPA-Induced Insomnia in Pentobarbital-Induced Sleep Test

**FIGURE 6:**
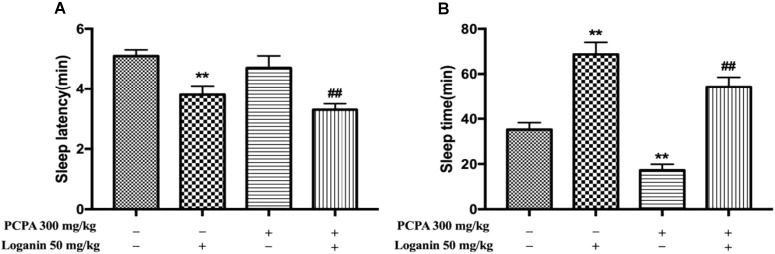
Effects of loganin on PCPA-induced insomnia in pentobarbital-treated mice. The sleep latency **(A)** and sleep time **(B)** were assessed. Data are presented as mean ± SEM (*n* = 10). ^∗∗^*P* < 0.01 vs. vehicle (Student-Newman-Keuls test); ^##^*P* < 0.01, vs. PCPA alone (unpaired Student’s *t*-test).

As shown in Figure 6, administration of PCPA could induce absolute insomnia by attenuating sleep time (^∗∗^*P* < 0.01, Figure 6B), but it had no effect on sleep latency. Loganin (50 mg/kg) remarkably decreased sleep latency (^∗∗^*P* < 0.01, Figure 6A) in the absence or presence of PCPA. Loganin (50 mg/kg) could also extend decreased sleep time induced by PCPA (^∗∗^*P* < 0.01, Figure 6B).

### Synergistic Hypnosis Effect of Low-Dose Loganin With 5-HTP in Pentobarbital-Induced Sleep Test

**FIGURE 7:**
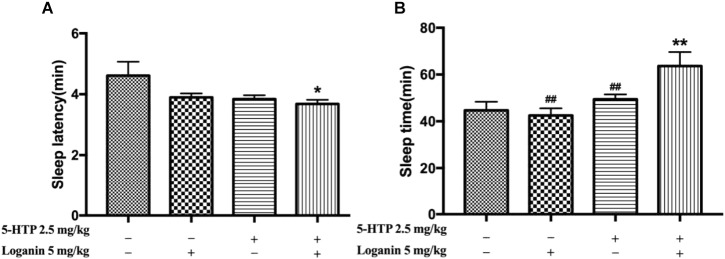
Synergic effects of loganin with 5-HTP on sleep in pentobarbital-treated mice. The sleep latency **(A)** and sleep time **(B)** were assessed. Data are presented as mean ± SEM (*n* = 12). ^∗^*P* < 0.05, ^∗∗^*P* < 0.01, vs. vehicle (Student-Newman-Keuls test); ^##^*P* < 0.01, vs. group treated loganin and 5-HTP (unpaired Student’s *t*-test).

Furthermore, low-dose loganin and 5-HTP was given individually or together in pentobarbital-induced sleep test. Neither loganin (5 mg/kg) nor 5-HTP (2.5 mg/kg) individually affected the sleep latency or sleep time induced by the hypnotic dose of pentobarbital (Figure 7A); however, coadministration of loganin (5 mg/kg) and 5-HTP (2.5 mg/kg) exhibited a synergistic effect of shortening sleep latency (^∗^*P* < 0.05, Figure 7A) and prolonging sleep time significantly (^∗∗^*P* < 0.01, Figure 7B). The coadministration significantly increased sleep onset (^∗∗^*P* < 0.01, Table 2) in mice treated with a subhypnotic dose of pentobarbital as well.

**Table 2:** Effect of loganin combined with 5-HTP on sleep onset of mice treated with hypnotic dosage of pentobarbital (45 mg/kg, i.p.).

Groups	Dosage (mg/kg)	No. of falling asleep/total	Sleep onset (%)
Vehicle	–	0/12	0.0
5-HTP	2.5 (i.p.)	2/12	16.7^∗^
Loganin	5 (i.g.)	2/12	16.7^∗^
5-HTP + Loganin	2.5 (i.p.) +5 (i.g.)	12/12	91.67^∗∗^

*The number of mice falling asleep was recorded (n = 12). ^∗∗^P < 0.01, vs. vehicle (χ^2^ test). ^∗^P < 0.05, vs. 2.5 mg/kg 5-HTP + 2.5 mg/kg loganin (χ^2^ test).*

### Sleep Architecture in Freely Moving Rats

**FIGURE 8:**
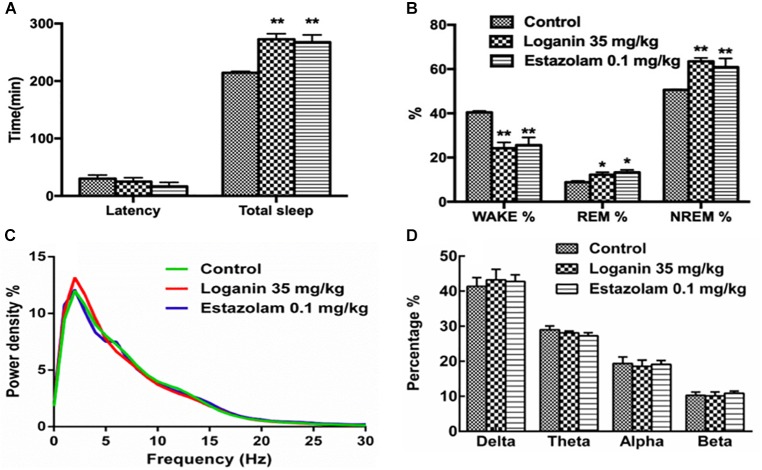
Effect of loganin on the sleep architecture in freely moving rats. EEG and EMG recordings were immediately applied 6 h after intragastric administration of loganin (35 mg/kg, i.g.) or vehicle. Total sleep and sleep latency **(A)**, percentage of wakefulness, REM sleep and NREM sleep **(B)**, percentage of power density **(C)** and percentage of delta (0.5–4 Hz), theta (4–8 Hz), alpha (8–13 Hz) and beta bands (13–30 Hz) of NREM sleep **(D)** were examined. Data are presented as mean ± SEM (*n* = 4–5). ^∗^*P* < 0.05, ^∗∗^*P* < 0.01, vs. vehicle (One-way ANOVA followed by Least Significant Difference).

The EEG and EMG recordings in freely moving rats showed that loganin clearly prolonged total sleep time (^∗∗^*P* < 0.01, Figure 8A). Loganin (35 mg/kg, i.g.) prominently increased the ratio of NREM sleep and shortened wakefulness (^∗∗^*P* < 0.01, Figure 8B), meanwhile, and it increased REM sleep (^∗^*P* < 0.05, Figure 8B) at 6 h after intragastric administration compared with the vehicle group. As the positive control group, estazolam also significantly increased NREM sleep and REM sleep (Figure 8B). In addition, the EEG power spectra showed that, similar to estazolam, loganin had almost no effect on the EEG power density of NREM sleep (Figure 8C,D), which is similar to estazolam.

### The Monoamine Neurotransmitter Levels in Hippocampus, Prefrontal Cortex, and Striatum of Mice

The result in the Sections “PCPA-induced insomnia” and “5-HTP-induced sleep” suggested that, neurotransmitter might be involved in the hypnotic activity of loganin. Therefore, the concentrations of monoamines and their metabolites in the mouse hippocampus, prefrontal cortex and striatum 1 h after loganin (35 mg/kg) treatment were measured. As shown in Table 3, in the prefrontal cortex, loganin significantly increased the ratio of 5-HIAA/5-HT, while decreasing the concentration of 5-HT, and simultaneously decreased the concentration of DA and its metabolite DOPAC. No changes in NE and 5-HIAA concentrations in the hippocampus, prefrontal cortex or striatum occurred after loganin treatment.

**Table 3:** The concentrations of NE, 5-HT, DA, and their metabolites in the hippocampus, prefrontal cortex and striatum after loganin treatment.

	Group	NE (ng/g)	5-HT (ng/g)	5-HIAA (ng/g)	DA (ng/g)	DOPAC (ng/g)	5-HIAA/5-HT	DOPAC/DA
**PFC**	Control	673.5 ± 119.1	1115 ± 151.4	337.5 ± 49.21	1031 ± 698.4	417.3 ± 145.4	0.29 ± 0.02	0.58 ± 0.18
	Loganin	469.5 ± 72.33	687.7 ± 91.16^∗^	241.3 ± 40.44	223.8 ± 56.61^∗∗^	124.7 ± 8.557^∗∗^	0.35 ± 0.05^∗^	0.63 ± 0.071
**STR**	Control	263 ± 64.29	466.8 ± 99.63	322.1 ± 73.21	16052 ± 3693	1088 ± 190.4	0.78 ± 0.14	0.09 ± 0.005
	Loganin	265.6 ± 26.61	723 ± 80.8	605.2 ± 92.07^∗^	15287 ± 1645	1284 ± 125.5	0.80 ± 0.07	0.09 ± 0.003
**HIP**	Control	478.4 ± 78.22	445.6 ± 61.47	346.8 ± 84.66	66.42 ± 16.19	ND	0.60 ± 0.10	ND
	Loganin	515.9 ± 68.09	499.3 ± 62.03	382.7 ± 98.16	75.44 ± 29.22	ND	0.59 ± 0.11	ND

*Data are presented as means ± SEM (n = 9). ^∗^P < 0.05, ^∗∗^P < 0.01, vs. control (unpaired Student’s t-test); ND: not detectable.*

### The Activation of GABAergic Neurons in VLPO of Rats

**FIGURE 9:**
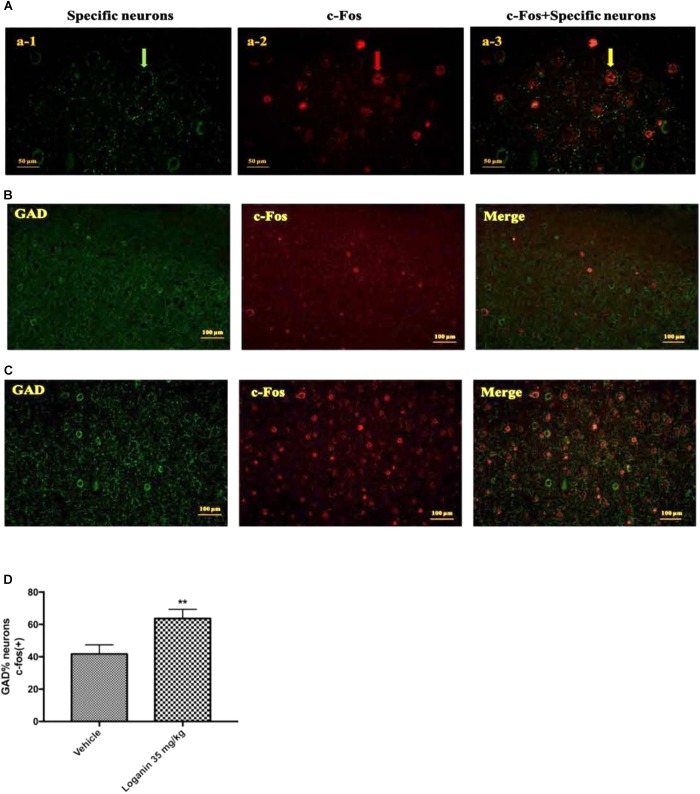
The effect of Loganin on c-Fos expression in the VLPO. The GABAergic neurons were labeled by green, c-Fos was labeled by red. **(A)** An example of photomicrographs of the double-immunostained neurons (scale bar = 50 μm; original magnification 400×). Fluorescence micrographs of c-Fos and GAD in normal mice **(B)** and mice treated with loganin 35 mg/kg **(C)** (scale bar = 100 μm; original magnification 200×). **(D)** Quantification of the number of c-Fos positive GABAergic neurons. Data are represented as mean ± SEM (*n* = 5). ^∗∗^*P* < 0.01, vs. vehicle (unpaired Student’s *t*-test).

To study the possible mechanism of the sleep-enhancing effects of loganin in rats, the activity of loganin in the VLPO sleep center was investigated. GAD is a marker for GABAergic cells. c-Fos expression is a cellular marker of neuronal activity. As shown in Figure 9, at 1 h after administration, loganin at a hypnotic dose of 35 mg/kg observably increased the ratio of c-Fos (+) in glutamic acid decarboxylase (GAD) (+) neurons in the VLPO (^∗∗^*P* < 0.01, Figure 9D). These results suggested that treatment with loganin significantly increased c-Fos expression in the GABAergic neurons of the VLPO (^∗∗^*P* < 0.01), as compared with the vehicle control.

## Discussion

Loganin, one of the major bioactive iridoid glycosides from Corni fructus, has been studied in many aspects; however, the sedative and hypnotic effects have not been reported. The present study showed that loganin significantly decreased locomotor activity in normal mice, potentiated the hypnotic effect of sodium pentobarbital. These results indicated that loganin might show central nervous inhibition activity.

Caffeine administration provides a simple and effective insomnia model, with difficulty in sleep initiation (Alexeev et al., 2012). In the present study, loganin could not promote the decreased sleep time induced by caffeine.

Previous studies have proved that 5-HT is the key regulator of sleep-wake. To investigate the role of 5-HT on the hypnotic activity of loganin, the effects of PCPA and 5-HTP were elevated. PCPA, a blocker of tryptophan hydroxylase, has been shown to inhibit more than 95% of 5-HT biosynthesis (Khanna et al., 1979). The effect of PCPA has been characterized by an initial sedative phase, which is followed by a phase of reduced sleep and a recovery phase (Borbély et al., 1981). 5-HTP, the precursor of 5-hydroxytryptamine (5-HT), prolonged pentobarbital-induced sleep time in a dose-dependent manner (Zhao et al., 2004). In our study, PCPA-induced insomnia could be recovered by loganin (50 mg/kg). Loganin (5 mg/kg) exerted synergistic hypnosis effect with 5-HTP, promoting sleep onset with a subhypnotic dose of pentobarbital and reducing sleep latency and prolonging sleep time induced by a hypnotic dose of pentobarbital in mice. Taken together, we supposed that serotonergic system might be involved in the mechanism underlying the hypnotic effects of loganin.

Neurotransmitters and their metabolites are known to play a significant role in the nervous system for numerous organisms. Changes in the metabolism and levels of monoamine neurotransmitters have also been associated with insomnia (Wei et al., 2014). Thus, we detected the effects of loganin on the levels of 5-HT, DA, NE, and their metabolites 5-HIAA, DOPAC, and HVA. Our results showed that loganin decreased the concentration of 5-HT, DA, and DOPAC, and increased the 5-HIAA/5-HT ratio in the prefrontal cortex. These results suggested that the serotonergic and dopaminergic neurons might be mainly involved in the mechanism of the hypnotic effects of loganin.

Mammalian sleep is composed of NREM and REM sleep alternation (Weber, 2017). Loganin significantly increased NREM sleep and total sleep, and remarkably shortened wakefulness. Similar to estazolam, loganin did not significantly reduce the EEG power of delta, which indicated that the EEG power following administration of loganin was similar to that of natural sleep. This suggests that loganin is compatible with physiological sleep. Therefore, loganin might be used for the treatment of insomnia.

Many studies have shown that neurons in the VLPO region show c-Fos activation after sleep and provide GABAergic innervation of the major monoamine arousal systems, which suggests that they may be a necessary part of the brain circuitry (Gallopin et al., 2000). It was reported that the VLPO cluster is a necessary component of sleep circuitry, without which NREM sleep is severely impaired. The discharge rate of VLPO neurons increases just before or at the transition from wakefulness to NREM sleep and decreases prior to the transition from NREM or REM sleep to wakefulness (Szymusiak et al., 1998). Results showed that loganin at a hypnotic dose of 35 mg/kg observably increased the ratio of c-Fos (+) in glutamic acid decarboxylase (GAD) (+) neurons in the VLPO, which indicated that the hypnotic effect of loganin may be mediated by activation of the sleep-promoting GABAergic neurons in the VLPO.

## Conclusion

In conclusion, this is the first study to confirm the sedative and hypnotic effects of loganin. Moreover, 5-HT, DA, and GAD activation might be involved in the mechanism of sleep-promoting effect and sleep architecture regulation of loganin. Other mechanisms involved in the sedative and hypnotic effects of loganin deserve more attention and further investigation. Taken together, these findings suggest that loganin might be a potential candidate drug for insomnia.

## Ethics Statement

This study was carried out in accordance with the recommendations of “National Institutes of Health Guide for the Care and Use of Laboratory Animals of China.” The protocol was approved by the “Animal Care Committee of China-Japan Friendship Hospital.”

## Author Contributions

J-KX and W-KZ designed the research and polished the manuscript. RS, YH, and H-YQ performed the experiments. T-LL provided the EGG and EMG instruments and experimental guidance. RS, YH, and YY drafted the manuscript. JH, W-WL, and C-YX revised the manuscript.

## Conflict of Interest Statement

The authors declare that the research was conducted in the absence of any commercial or financial relationships that could be construed as a potential conflict of interest.
